# Designing for Clinical Change: Creating an Intervention to Implement New Statin Guidelines in a Primary Care Clinic

**DOI:** 10.2196/humanfactors.9030

**Published:** 2018-04-24

**Authors:** Melissa DeJonckheere, Claire H Robinson, Lindsey Evans, Julie Lowery, Bradley Youles, Adam Tremblay, Caitlin Kelley, Jeremy B Sussman

**Affiliations:** ^1^ Department of Family Medicine University of Michigan Ann Arbor, MI United States; ^2^ Center for Clinical Management Research Veterans Affairs Ann Arbor Healthcare System Ann Arbor, MI United States; ^3^ Department of Internal Medicine University of Michigan Ann Arbor, MI United States; ^4^ General Medicine Veterans Affairs Ann Arbor Healthcare System Ann Arbor, MI United States; ^5^ Institute for Healthcare Policy and Innovation University of Michigan Ann Arbor, MI United States

**Keywords:** cardiovascular disease, preventative medicine, clinical decision support, user-centered design, qualitative research, implementation

## Abstract

**Background:**

Recent clinical practice guidelines from major national organizations, including a joint United States Department of Veterans Affairs (VA) and Department of Defense (DoD) committee, have substantially changed recommendations for the use of the cholesterol-lowering statin medications after years of relative stability. Because statin medications are among the most commonly prescribed treatments in the United States, any change in their use may have significant implications for patients and providers alike. Prior research has shown that effective implementation interventions should be both user centered and specifically chosen to address identified barriers.

**Objective:**

The objectives of this study were to identify potential determinants of provider uptake of the new statin guidelines and to use that information to tailor a coordinated and streamlined local quality improvement intervention focused on prescribing appropriate statins.

**Methods:**

We employed user-centered design principles to guide the development and testing of a multicomponent guideline implementation intervention to improve statin prescribing. This paper describes the intervention development process whereby semistructured qualitative interviews with providers were conducted to (1) illuminate the knowledge, attitudes, and behaviors of providers and (2) elicit feedback on intervention prototypes developed to align with and support the use of the VA/DoD guidelines. Our aim was to use this information to design a local quality improvement intervention focused on statin prescribing that was tailored to the needs of primary care providers at our facility. Cabana’s Clinical Practice Guidelines Framework for Improvement and Nielsen’s Usability Heuristics were used to guide the analysis of data obtained in the intervention development process.

**Results:**

Semistructured qualitative interviews were conducted with 15 primary care Patient Aligned Care Team professionals (13 physicians and 2 clinical pharmacists) at a single VA medical center. Findings highlight that providers were generally comfortable with the paradigm shift to risk-based guidelines but less clear on the need for the VA/DoD guidelines in specific. Providers preferred a clinical decision support tool that helped them calculate patient risk and guide their care without limiting autonomy. They were less comfortable with risk communication and performance measurement systems that do not account for shared decision making. When possible, we incorporated their recommendations into the intervention.

**Conclusions:**

By combining qualitative methods and user-centered design principles, we could inform the design of a multicomponent guideline implementation intervention to better address the needs and preferences of providers, including clear and direct language, logical decision prompts with an option to dismiss a clinical decision support tool, and logical ordering of feedback information. Additionally, this process allowed us to identify future design considerations for quality improvement interventions.

## Introduction

### Background

There has been a dramatic shift with respect to how guidelines recommend that American medical providers should prescribe commonly used cholesterol-lowering statin drugs [1]. In 2013 and 2014, the American College of Cardiology (ACC) and American Heart Association (AHA) and the United States Department of Veterans Affairs (VA) and Department of Defense (DoD) released new clinical practice guidelines on the treatment of blood cholesterol to reduce cardiovascular risk in adults [2-4]. Where previous guidelines had focused on giving increasing doses of statins until a patient’s cholesterol level dropped below a specific target, both new guidelines recommend fixed doses of medicine based on the patient’s atherosclerotic cardiovascular disease (ASCVD) *risk*, the chance that the patient will develop cardiovascular disease (CVD) [2,5-9].

The new guidelines present challenges to adoption. First, moving away from cholesterol target-based treatment models represents a conceptual change in clinical practice. Second, in many cases, risk-based guidelines would require providers to calculate a patient’s ASCVD risk, which could substantially alter a provider’s workflow. Third, the discrepancies between the VA/DoD and ACC/AHA guidelines could cause confusion [10].

Effective implementation of new guidelines should recognize the existing barriers to adoption [11], including providers’ knowledge, attitudes, and behaviors about those guidelines [12]. They must also address those barriers in ways that are effective, accurate, and user centered. Existing strategies, including provider education, clinical decision support, and audit and feedback must address the barriers and the providers’ needs [12]. This requires a strong framework for designing an intervention and for making sure the intervention is effective.

Effective interventions fit the needs of the end users. To this end, user-centered design focuses on understanding the physiological, cognitive, and social aspects of the intended user that could alter how someone will use a tool or system [13]. In a health care setting, user-centered design can be employed to create or adapt tools that are consistent with the physiological, cognitive, and social needs of providers to address challenges to adoption and increase the likelihood of their use.

### Objectives

In this study, we developed and tested a multicomponent guideline implementation intervention (hereafter referred to as the intervention) to improve statin prescribing. Our intervention was developed with semistructured qualitative interviews, an established theoretical framework, and principles of user-centered design. This paper describes the intervention development process with providers, which was conducted to (1) illuminate knowledge, attitudes, and behaviors and (2) elicit feedback on intervention prototypes developed to align with and support the use of the VA/DoD guidelines. Our aim was to use this information to design a local quality improvement intervention focused on statin prescribing that was tailored to the needs of primary care providers.

## Methods

### Intervention Background and Development

We sought to develop and test a multicomponent guideline implementation intervention to improve statin prescribing. The processes were designed to find new, but practical, components for the intervention and help us improve the processes that we already had planned based on the literature and existing practices. For example, research demonstrates that a multicomponent intervention is often more effective than a single approach alone [14].

In the following section, we describe the components of the intervention: educational program, clinical decision support tool, and performance measurement with audit and feedback. Paper-based prototypes were created as working models to be tested for acceptability before investing in computerized systems. The prototypes were modeled after currently existing tools utilized in the VA health system to facilitate providers’ ability to imagine how the prototypes would function in their current workflow. In our user-centered design process, we asked providers to identify their needs and preferences specific to the 4 prototypes described below.

#### Educational Program

In collaboration with providers, we developed an educational program about the new clinical practice guidelines to be delivered to all providers before the intervention began. The educational program lasted 15 min. It included a summary of the guidelines and 3 cases that demonstrated the differences between the new guidelines and the old. We also developed a single-page tool describing and comparing the VA/DoD and the ACC/AHA Clinical Practice Guidelines (see Multimedia Appendix 1). The single-page tool was designed to be a concise and convenient reminder of changes to the statin guidelines.

#### Clinical Decision Support Tool

The clinical decision support tool was designed to address 2 predicted quality gaps—the traditional reminder role of pop-ups and a need to make it easier to follow the guidelines. The new guidelines require providers to calculate the risk of ASCVD for some patients using an algorithm that incorporates risk factors (eg, age, sex, smoking, high-density lipoprotein cholesterol, total cholesterol, systolic blood pressure), which significantly complicates use of the guidelines. In our facility, an ASCVD risk algorithm has not yet been incorporated into the electronic medical record, and providers access ASCVD risk calculators through external websites. The paper-based prototype resembled the existing clinical decision support alerts, thus meeting the reminder role and automatic calculations of ASCVD risk of computerized clinical decision support (see Multimedia Appendix 2). The impact of not having the tool in the electronic health record was evaluated in our interviews.

#### Performance Measurement With Audit and Feedback

Traditionally, performance measurement is used in pay-for-performance programs within the VA. We worked with the VA’s Center for Analytics and Reporting to create a novel performance measure that is aligned with the VA/DoD guidelines (hereafter referred to as the VA proposed performance measure; see Multimedia Appendix 3). In the VA proposed performance measure, providers would have stronger incentives for patients for whom treatment is more likely to be clinically important using a weighted point measurement system to create risk categorization of patient groups. For example, the VA proposed performance measure would award providers different points for prescribing a moderate dose statin to a patient with clinical ASCVD (5 points), a patient with diabetes (3 points), and a patient with a 10-year ASCVD risk greater than 12% (1 point). In distinction, other performance measures, such as those of the Healthcare Effectiveness Data Information Set, do not incorporate risk prediction in patient treatment recommendations. The weighted VA proposed performance measure was designed to emphasize prevention through risk calculation.

We designed an audit and feedback report template (see Multimedia Appendix 4), wherein providers would be informed of their individual performance on the VA proposed performance measure. The template includes 2 provider performance reports. The first includes breakdown of provider performance by patient risk categorization (eg, history of ASCVD; diabetes; low-density lipoprotein, LDL >190; high risk; low risk). The second displays performance by overall statin use across patients. Similar to the VA proposed performance measure, the audit and feedback report features risk prioritization.

### Setting and Participants

This local quality improvement project was conducted in primary care at a single Veterans Affairs Medical Center (VAMC) between late October 2015 and June 2016. In total, 37 professionals across 5 Patient Aligned Care Teams (PACTs)—including 32 physicians with their own patient panel at the start of the project and 5 pharmacists—were invited to participate in qualitative interviews via email. Though 37 professionals were invited, data collection was designed to continue until thematic saturation was reached [15-17]. Invitees were presented with a project information sheet at the time of initial email contact, which was reviewed at the time of the interview.

### Ethical Considerations

Veterans Health Administration (VHA) Handbook 1058_05 [18] provides guidance about authorization of manuscripts that have been developed through nonresearch activities (ie, without institutional review board approval under the authority of VHA operations). All VHA authors of this manuscript attest that the activities that resulted in producing this manuscript were not conducted as part of a research project but as part of the nonresearch evaluation conducted under the authority of the VA’s Quality Enhancement Research Initiative.

### Data Collection

We conducted semistructured interviews with providers to guide the development and testing of a multicomponent guideline implementation intervention. A qualitative approach was selected to explore user knowledge, attitudes, and behavior to improve the adaptation and implementation of the intervention. Interviews were audiotaped, transcribed verbatim, and lasted an average of 49 min. One member of the research team (CR) conducted all interviews while a research assistant took notes. Providers were not compensated for their time, and participation was completely voluntary. We began by eliciting feedback on determinants of providers’ guideline uptake. We then engaged providers in a user-centered design process to examine and improve prototypes for the 4 components of the intervention.

### Analysis

After a review of the literature, we determined that existing frameworks could be used to understand implementation of clinical guidelines in our setting. We used in-depth qualitative research principles structured by the Clinical Practice Guidelines Framework for Improvement [12] to guide our understanding of the barriers and facilitators to use and Nielsen’s Usability Heuristics [19] to guide the user-centeredness of our development process. The Clinical Practice Guidelines Framework for Improvement examines individual-level factors (knowledge, attitudes, and behaviors) of providers [20], whereas Nielsen focuses on elements of user-centeredness and design.

Using an initial codebook based on constructs from the Clinical Practice Guidelines Framework for Improvement and Nielsen’s Usability Heuristics (see Table 1), we (JS, CR, and MD) used a deductive approach to apply descriptive codes to 3 transcripts and modified our codes based on the data. We then applied codes from the modified codebook to 3 more transcripts and discussed our codes to determine consensus. The remaining 9 transcripts were coded by 1 team member (MD). We used QSR International’s NVivo version 11 data analysis software to apply codes to segments of text and to create code reports that grouped all text sharing the same code. Code reports were then summarized independently by the project team members (JS, CR, MD) and discussed to reach shared understanding of themes.

Project team members (JS, CR, CK, and BY) discussed interview notes in team meetings. Following a user-centered design approach, the team discussed provider needs related to the intervention and made changes to the wording and format of the prototypes as interviews progressed. When there was misalignment between providers’ preferences and design decisions, we used an adapted consensus process [21] to decide which suggested modifications were feasible.

**Table 1:** Initial codebook incorporating individual-level factors and elements of design.

Code		Definition
**Guideline factors**		
	Familiarity	Awareness/knowledge/use of the guidelines
	Self-efficacy	Ability to follow the guideline
	Expected efficacy	Will improve clinical outcomes for patients (prevent heart attacks and strokes)
	Previous practice	Change from previous care? How much does changing care affect the provider?
	Use of guidelines in general	Motivated by/trust/use of external guidelines in general
	Accept/reject guidelines	Agreement/disagreement with new guidelines
	Risk-benefit comparison	How do the benefits to patient/outcomes compare to the risks of implementing guidelines
	Evidence-based	Perception that guidelines are consistent with evidence-based practice (credibility)
	Oversimplified cookbook	Concerned that the guideline is too regimented, missing real-world nuance
	Autonomy	Effect on autonomy
	Standardization of practice	Makes it so all providers provide similar care
	Teamlet role/responsibility	Role of nursing, pharmacy, other staff in patient adherence to statins
	Clarity	Ability to understand the guidelines
	Gaming	Activity that produces apparent change in the measure, but no genuine change in the underlying performance
**Patient factors**		
	Patient resistance	Willingness of patients to take medications, engage in conversation, accept recommendations
	Patient tolerance	Side effects of medication prohibit adherence
	Shared decision making	Effect of guidelines on shared decision making
**Provider factors**		
	Clinical influences	Who influences uptake? Professional role, individual respect, professional, and/or personal interactions?
	Performance pay	Does reimbursement or performance pay alter uptake?
	Performance measurement system	Agreement with use of performance measurement system
	Audit and feedback-Pt-level feedback	Use of fallout reports with specific patients to target/follow up with
	Communication with patients	Strategies or tools for effective communication with patients
**Practice setting factors**		
	Reminder system (decision support tool)	Need for a reminder system for ease of use, understanding, calculation, etc
	Catch missed patients	Tool helps recognize who would benefit
	# of clinical reminders	Amount of clinical reminders seen by providers
	Provider education	Educational resources, strategies, tools for providers
	Not applicable to practice population	Relevance of guidelines to practice
	Not practical in our setting	Would require unavailable technology, nonformulary medicines, or unavailable specialists
	Insufficient staff or support	Ability of practice to use guidelines with existing staff resources
	Practicality/prioritization	Time to address guideline, fit with workflow
**Usability heuristics**		
	Transparency of calculation	Provider understands how the recommendation was determined
	Autonomy/allows complexity	Allows for and explains provider choices (eg, emergency exit)
	Accuracy	Are the recommendations correct (by what they intend to have)
	Cognitive ease of use	Saves or creates providers the need to think, calculate, remember
	Speed/ease of use	Time-consuming/saving, fits workflow

## Results

### Participant Description

In total, 15 individuals—13 physicians and 2 clinical pharmacists—participated in interviews and represented all 5 PACT teams. In total, 9 providers did not respond to 3 email invitations and 13 declined to participate. There were no observable differences in gender, age, or participation in the educational seminar, between those we interviewed and those who did not participate.

### Summary of Findings

Overall, providers were generally comfortable with the paradigm shift to risk-based guidelines but less clear on the need for the VA/DoD guidelines in specific. They preferred tools that helped them provide the care they wanted to provide without limiting their autonomy (see Table 2 for abbreviated list of changes made in response to interviews; see Multimedia Appendix 5 for detailed list of user-centered design changes).

### Guidelines

#### Providers Accept the Paradigm Shift in Cholesterol Treatment but Some Question the Need for Separate Department of Veterans Affairs/Department of Defense (VA/DoD) Statin Guidelines

Most providers felt the risk paradigm was more closely aligned to their clinical perspective:

We’ve moved away from focusing on LDL, this one just seems more compelling…here’s the person’s risk, it just seems more informative and like a compelling reason to treat.

Participant #13

Others highlighted the benefit of providing patients with more precise, tailored risk estimates using risk-based guidelines.

One core distinction between the guidelines is that the VA/DoD guidelines are generally less aggressive than the ACC/AHA guidelines: they recommend treatment for fewer people, permit use of less-intense statin regimens, and create a gray zone where treatment is neither recommended for nor against. A few providers stated their preference for the VA/DoD guidelines and felt the ACC/AHA guidelines encouraged overtreatment. One provider explained:

There may be some people that are jumping right to high potency when that's not necessary, especially in the elderly population which we have a ton of.

Participant #9

Another participant said:

[I’m] not sure of the distinction between the AHA guidelines and these [VA/DoD] guidelines.

Participant #8

Several providers did not recognize the need for separate VA guidelines at all. One of the participants admitted:

...most of my colleagues here have kind of adapted it [the VA/DoD guidelines].

Participant #15

A few developed approaches that incorporated aspects of both sets of guidelines, such as one who appreciated the deemphasis of routine cholesterol monitoring in the VA/DoD guidelines but preferred the risk cut points established in the ACC/AHA guidelines.

Due to their patient population, a few providers noted that the differences between the 2 guidelines would likely have a very small impact:

They all have diabetes, many of them smoke, and they all have hypertension. A lot of them already have cardiovascular disease, so you’re not really even doing a risk assessment. Many of them don’t specifically fall into the scope of this, so to be honest I haven’t used the VA one much just because there’s not been much need for it in the patients that I see.

Participant #7

#### Within the Risk Paradigm, Providers Are Not Confident in How to Deal With Shifting Risk

Providers were generally comfortable with the role of risk prediction in the guidelines. They did express some confusion about how to address changing risk factors and the lack of consistency of risk prediction. For these problems, they felt that the guidelines were not responsive. One provider explained:

I think the calculators can vary a lot, depending on what someone’s blood pressure is that day or their smoking status. Those kinds of things can change. Then someone if they quit smoking might not be, you know, the same risk as they were 10 minutes ago...So, I think it’s not exactly clear cut...

Participant #14

#### The Paradigm Shift Creates New Responsibilities in Doctor-Patient Communication

Several providers felt that their patients might find it difficult to shift away from cholesterol treatment targets. Patients are familiar with recommendations to improve their cholesterol numbers. As one provider explained:

I think there is still a little bit of resistance. Patients are really caught up on the LDL number because, I guess we used to really drive that hard, like “Oh, your LDL should be this and it’s too high and so we’re going to add these other drugs, or increase the dose,” or whatever it might be. I think some people were still really hung up on those numbers.

Participant #7

Comparatively, risk reduction is more “abstract” than LDL reduction. Several providers described that patients “like to see that [the treatment is] doing something,” which is difficult to demonstrate under guidelines that do not specifically call for routine cholesterol monitoring. Thus, providers were concerned about nonadherence:

I worry that we are going to have even more trouble initiating and getting people to adhere to statins when we are talking about them in this new kind of abstract confusing way for patients. I have probably not been as aggressive in moving towards these newer guidelines in part for that reason.

Participant #2

**Table 2:** Abbreviated list of user-centered design considerations for intervention components.

Tool and user suggestion		Impact on adoption
**Clinical decision support design**		
	Include high/medium/low-risk language in reminder—facilitates conversation with patient	Implemented
	Disable reminder for patients receiving palliative care	Implemented
	Prepopulate risk score automatically within reminder	Future consideration
	Alert only when appropriate (disable reminder for patients with complicated clinical situations)	Future consideration
	Add specific risk percentage in reminder rather than high/medium/low language	Not used
	Add additional line for comments	Not used
**Audit and feedback design**		
	Organize patient fallout by risk category	Implemented
	Clarify provider comparison group (local vs Department of Veterans Affairs)	Future consideration
	Devise mechanism/algorithm that accounts for complicated patients in performance measure and subsequently in the audit and feedback report	Future consideration
	Provide credit for shared decision making	Future consideration
	Include specific and actionable performance improvement suggestions	Future consideration
	Remove provider percentile altogether because it creates undue angst	Not used

### Clinical Decision Support Tool

#### Providers Desire Clinical Decision Support Tools That Allow for Cognitive Ease of Use and Speed

Providers’ interest in having a clinical decision support tool during the patient encounter was based around efficiency:

If the reminder already calculated the risk, I’d love that. I hate having to go to the internet, or look on my smartphone, so I think the ideal reminder would calculate the risk for you.

Participant #6

A few providers indicated that the clinical decision support tool may be especially useful in patients whom the calculator estimates to be at high risk for ASCVD but have no history of heart attack or stroke:

In this particular case, I like it because this is one that may not jump out immediately at you. This person doesn’t have coronary disease so it’s kind of helping you work through and reminding you where the guidelines stay.

Participant #9

#### Providers Want Clinical Decision Support Tools That Allow for Autonomy

When asked about the computerization of clinical decision support tools, most providers indicated a need for autonomy within the system, whereby providers can exit or cancel a clinical reminder when it is inappropriate or inaccurate for the particular visit or patient:

Sometimes it seems like things come up that aren’t supposed to, or they don’t come up and they should…I think there’s often circumstances where it’s like, “How do you get out of this loop?” where this isn’t right and it should go away, but you can’t make it go away and so I like that there’s an option for like, “This is wrong,” and so you can get out of that.

Participant #7

Providers said they generally appreciate being reminded when a patient is not meeting a guideline but want to be able to accurately state why the patient is not on a statin rather than bend the truth simply to disable the reminder.

#### Providers Want Clinical Decision Support Tools That Can Be Disabled

Providers wanted a clinical decision support tool that would not continue to alert after an issue has been addressed. However, there was some debate as to which clinical situations should lead to a reminder being disabled indefinitely and which would warrant a revisited conversation:

If you had a discussion with the patient and they decided against it, okay, if you had a discussion with the patient and they decided for it, okay. I’d never not do it because they were poor in the past, you know, we’d have a discussion and in that moment, I’d give them every opportunity to say they’re going to try it. So, I would never let the history of non-adherence stop me from providing it unless they actively told me.

Participant #9

### Performance Measurement With Audit and Feedback

#### Some Providers Prefer Dichotomous Performance Measures, Whereas Others Prefer Performance Measures That Incorporate Risk Categorization of Patient Groups

The team proposed a new performance measure consistent with the new guidelines that would provide weighted performance assessment. In this system, patients for whom statin treatment was particularly likely to prevent a heart attack or stroke would be given more credit in evaluation. Providers had mixed feelings about the proposed VA performance measure, particularly the idea of weighting the performance measure to reflect risk categorization of patient groups (based on patients’ ASCVD risk):

So, I could tell you with the measures, I will be honest with you; I don’t like the idea of weighted. I like either you made it or you didn’t…and I think either you’re treating them appropriately or you're not…

Participant #4

Other providers preferred the proposed VA performance measure and that having a measure that “reflects” that difference may improve care:

...overall risk for some of these patients is higher or lower depending on which of these [risk] categories they fall into.

Participant #7

#### Providers Want More Credit for Shared Decision Making

Several providers were concerned with the lack of credit given for shared decision making in the proposed and existing VA performance measures. Most providers agreed that the high-risk patients, or the “no-brainers” as one provider put it, take less effort and time to convince to initiate and adhere to treatment because their risk is more palpable. Rather, it is the patients who:

feel fine and they haven’t had any negative outcomes yet [sometimes] are the hardest ones to get to comply and understand, educate about what’s in their best interest...

Participant #9

One provider specifically made the connection between the way pay-for-performance structures are designed and the lack of consideration given to shared decision making:

[Patients that fall in the intermediate risk category] You use a lot of energy with and you’re really not capturing that much value from the standpoint of, whatever it’s going to be, an A or money or whatever it is at the end that you get as your carrot. I don’t know how you would do it any other way that I think makes sense. I don’t think most of us are in it for the A or the money.

Participant #12

#### Providers Feel That Hierarchical Patient-Level Feedback Is Most Useful Within Audit and Feedback Reports

Providers regularly receive audit and feedback of their care within this clinic, usually in the form of printouts of tables of care provided. We attempted to understand how the new guidelines might alter the best way to provide audit and feedback. Providers generally preferred the audit and feedback report when broken down into component parts, indicating first, how the provider fares on each individual goal (ie, the percentage of the provider’s patients with ASCVD that are on a moderate or high-dose statin) and second, broken down by patient fallout, with the highest risk patients listed first, and the lower-risk patients listed last. Providers indicated that listing out patients that did not meet the guideline by risk category would be more actionable than having a single list of patients not meeting guidelines, as members of the PACT team would then be better able to triage follow-up phone calls. As one provider explained:

It does help you gauge again from the standpoint of, where do you least want to make mistakes, with the people that have significant disease already and if you had someone with very, very low risk taking a statin, it’s not going to be the worst thing in the world. I mean, you’re not happy about it, but I think that is important to see the breakdown.

Participant #9

Several individuals indicated that comparing providers by their percentile of measures met is not motivating, in part, because it can be difficult to distinguish who they are being compared with, whether it is providers at the local level, or providers at the system level. Another provider mentioned that delayed receipt of the report also decreases impact on provider behavior, stating:

I think there is a big enough disconnect between the guidelines and the results coming out of it.

Participant #12

#### Providers Would Value Audit and Feedback More if It Were Used to Help Their Care More Directly

Participants generally wanted performance measures and audit and feedback reports to be more tightly linked to ways to help the providers improve practice in response:

I want the researchers or whoever’s pulling this out for me, if somebody’s in the highest percent I want you to interview them and tell me what...are they doing to be in that percentile. I’m not kidding you...Clearly somebody’s in the top percentile. What are they doing?...It’s like, don’t just tell me where I’m at, tell me how to be better and do that by using this to find out who’s doing better...

Participant #4

Providers wished there would be a greater commitment toward teaching them about new guidelines and changes in practice, particularly those moving toward incorporating patient risk and shared decision making. On the basis of interviews, we found that providers are willing to adopt risk-based clinical guidelines and accompanying components if they are designed with care and are presented to providers in a clear and useful manner:

It’s more than just flipping on a switch and having some PowerPoint slides. I think that you really need to help clinicians move towards that, help them understand it, give them some strategies, give them some confidence for how to move in that direction because...it’s another paradigm shift that we need to be making, but I think we need help in order to get there.

Participant #2

## Discussion

### Principal Findings

We developed a system to create a multicomponent implementation intervention that was to be user-centered and evidence based. Our system helped us identify ways to improve aspects of the intervention and develop new ones (see Table 2).

**Design considerations for future interventions.**

Performance improvement suggestions

- **Representative quote:** *“So, don’t just tell me these are wrong, tell me what I need to do, give me useful information so I now know, ‘Oh, I need to call that patient and double it,’ versus whatever, I mean, just to say they’re on 40, that doesn’t mean anything to me. So, that’s the key thing, to make this helpful tell me what I need to do, because honestly, the more I know what I need to do, the more I can hand this to my nurse and say, ‘Hey! Call that patient, order a blood draw, tell him what it is, I’m going to change their med,’ make it so I don’t have to do anything, yeah, there we go.”* [Participant #4]
- **Proposed response:** Include specific and actionable performance improvement suggestions

Provider comparison group

- **Representative quote:** *“It tells me my provider percentile; I can never tell if it’s VA or local, okay so that’s part of it, so that’s one thing I would want to know VA or local.”* [Participant #12]
- **Proposed response:** Clarify provider comparison group

Patient fallout organization

- **Representative quote:** *“So, if you are going to work through a list, you want to start from the top and work your way down kind of deal. So, I think that that would be helpful because you’re, at least at the outset, you’re going to identify the most important areas for intervention…So, I do think that’s helpful from just like a time management perspective, like start here and then over the next six months we’ll get through everybody, but at least we’ll start at the top and work down to the people who are maybe less of a priority as far as you know statin and cardiovascular risk reduction.”* [Participant #7]
- **Proposed response:** Organize patient fallout by risk category

Accounting for complicated patients

- **Representative quote:** *“It depends if we give credit for having...if we could include documented adverse drug reaction to giving you credit, then that would be good or just taking those people out altogether, you know, so they’re not even in the, they’re not even in the denominator, um because you know, there are a lot of people who have statin, and this is where it’s provider, you know, it is provider. If you don’t ask and you don’t know, and you just keep pounding someone with statin and they’re feeling miserable, it’s not the right thing to do. So, if you’re not aware of the potential side effects or you’re not asking and you’re not dealing with it, then your numbers may look better but you may not be doing the patient a service. So, I would say if the goal includes, if you get credit for at least a documented adverse drug reaction, then I’d be fine with those numbers. If not, they need to come out of the denominator, if not, the goal needs to be a little bit lower or I would recommend it be lower.”* [Participant #9]
- **Proposed response:** Think of mechanism to account for complicated patients in performance measure and subsequently in the audit and feedback report

In short, we found that providers were interested in changing their care but needed support in doing so.

Our team incorporated feasible design suggestions into the prototype intervention, particularly when there was general agreement among providers on a given design element and it aligned with design and user experience best practices. Consistent with previous research [22,23], providers overwhelmingly preferred simple information, clear and accurate decision prompts, and logical ordering of information that aligned with their values and needs, such as including highest risk patients first on audit and feedback report fallout lists. More specifically, providers wanted to be able to accurately and rapidly use clinical decision support tools during the patient encounter without any loss to their autonomy [24]. Many of these wording or formatting suggestions were addressed in the second iteration of the clinical decision support tool and audit and feedback report template.

Some providers found the shift to new guidelines difficult, even when the guidelines were more closely aligned to their clinical perspective. For example, providers also felt guidelines don’t recognize the most difficult aspects of their work, particularly the time and resource demands of shared decision making and introducing the concept of risk, which is strongly emphasized in the new guidelines [25,26]. In addition, providers requested more evidence, education, and resources to make any clinical change. Educational and training resources for both providers and patients were thought to be essential in effective shared decision making and, as a result, adherence to statin guidelines. In response, we implemented an educational seminar during a primary care meeting whereby differences between the guidelines were highlighted by way of a pocket guide [27] and explained in detail before the commencement of the intervention phase of the project.

Our work adds to, but is supported by, existing research in implementation science on guideline implementation and how to change clinician habits. Our findings align well with our underlying framework, the Clinical Practice Guidelines Framework for Improvement [12]. As that framework and other research suggests, we found barriers and wide variation in providers’ knowledge, attitudes, and behaviors about the new guidelines [12,28]. Previous work has also found that providers find guidelines and performance measures demotivating, especially when they are not user-centered or well-aligned with the providers’ goals of care [29,30]. Similarly, decision support tools regularly impact patient care but details of usability also have large effects on provider satisfaction and uptake [31,32]. Our work is one of a few studies that have attempted to synthesize these diverse fields of research into a single intervention. Our findings were also unusual in noting the central divide between providers’ desire for new guidelines for support and efficiency versus a sense that they are intended to remove providers’ autonomy.

### Limitations and Future Research

We sampled a small number of providers from one VAMC. Nonetheless, the providers who participated in the interviews for this project provided important insights that influenced both the type and content of the intervention later executed at this site. We expect our research design to be transferable to other sites, as user-centered design and qualitative methods both emphasize local context.

We were also limited in our ability to incorporate many of our findings into the intervention. At times, providers’ opinions and preferences were at odds. Thus, our team needed to prioritize and rank feedback, accommodating feasible design suggestions with strong provider consensus, and vetoing design elements that were too provider-specific, acknowledging that a provider-specific interface is not feasible within the health system. Relatedly, there were requests for user-friendly features that were technologically infeasible. Consequently, we have identified future design considerations for each of the above domains (see Textbox 1) that were outside the scope of this project but could be considered in other projects.

Finally, the purpose of this study was to follow a user-centered design approach to capture the needs and preferences of providers in the final intervention design. Though beyond the scope of this study, future research should examine the effectiveness of similar multicomponent implementation interventions.

### Conclusions

The guideline implementation planning process provided important insights about the refinement of the intervention plan. By combining qualitative methods and user-centered design principles, we could understand the needs and preferences of providers and modify prototypes to increase their acceptability and usability in practice. Our findings allowed us to target several factors providers reported as being important determinants to the uptake of and adherence to clinical practice guidelines. The qualitative process of working with providers also allowed us to identify future design considerations for multicomponent guideline implementation interventions.

## Appendix

Multimedia Appendix 1 Single-page VA/DoD and ACC/AHA Clinical Practice Guidelines educational tool.

Multimedia Appendix 2 Clinical decision support tool.

Multimedia Appendix 3 VA proposed performance measure.

Multimedia Appendix 4 Audit and feedback template.

Multimedia Appendix 5 User-centered design changes to intervention components.

## Abbreviations

ACC: American College of Cardiology
AHA: American Heart Association
DoD: Department of Defense
PACT: Patient Aligned Care Team
VA: Department of Veterans Affairs
VAMC: Veterans Affairs Medical Center
VHA: Veterans Health Administration

## References

[1] Centers for Disease Control and Prevention.

[2] Goff Jr DC, Lloyd-Jones DM, Bennett G, Coady S, D'Agostino RB, Gibbons R, Greenland P, Lackland DT, Levy D, O'Donnell CJ, Robinson JG, Schwartz JS, Shero ST, Smith Jr SC, Sorlie P, Stone NJ, Wilson PW, Jordan HS, Nevo L, Wnek J, Anderson JL, Halperin JL, Albert NM, Bozkurt B, Brindis RG, Curtis LH, DeMets D, Hochman JS, Kovacs RJ, Ohman EM, Pressler SJ, Sellke FW, Shen WK, Smith SC, Tomaselli GF, American College of Cardiology/American Heart Association Task Force on Practice Guidelines (2014). 2013 ACC/AHA guideline on the assessment of cardiovascular risk: a report of the American College of Cardiology/American Heart Association Task Force on Practice Guidelines. Circulation.

[3] Downs JR, O'Malley PG (2015). Management of dyslipidemia for cardiovascular disease risk reduction: synopsis of the 2014 U.S. Department of Veterans Affairs and U.S. Department of Defense clinical practice guideline. Ann Intern Med.

[4] Bibbins-Domingo K, Grossman DC, Curry SJ, Davidson KW, Epling Jr JW, García FA, Gillman MW, Kemper AR, Krist AH, Kurth AE, Landefeld CS, LeFevre ML, Mangione CM, Phillips WR, Owens DK, Phipps MG, Pignone MP, US Preventive Services Task Force (2016). Statin use for the primary prevention of cardiovascular disease in adults: US Preventive Services Task Force Recommendation Statement. J Am Med Assoc.

[5] Hayward RA, Krumholz HM, Zulman DM, Timbie JW, Vijan S (2010). Optimizing statin treatment for primary prevention of coronary artery disease. Ann Intern Med.

[6] Sussman J, Vijan S, Hayward R (2013). Using benefit-based tailored treatment to improve the use of antihypertensive medications. Circulation.

[7] Hayward RA, Hofer TP, Vijan S (2006). Narrative review: lack of evidence for recommended low-density lipoprotein treatment targets: a solvable problem. Ann Intern Med.

[8] Krumholz HM, Hayward RA (2010). Shifting views on lipid lowering therapy. Br Med J.

[9] Sussman JB, Vijan S, Choi H, Hayward RA (2011). Individual and population benefits of daily aspirin therapy: a proposal for personalizing national guidelines. Circ Cardiovasc Qual Outcomes.

[10] Hendrani AD, Adesiyun T, Quispe R, Jones SR, Stone NJ, Blumenthal RS, Martin SS (2016). Dyslipidemia management in primary prevention of cardiovascular disease: current guidelines and strategies. World J Cardiol.

[11] Kortteisto T, Kaila M, Komulainen J, Mäntyranta T, Rissanen P (2010). Healthcare professionals' intentions to use clinical guidelines: a survey using the theory of planned behaviour. Implement Sci.

[12] Cabana MD, Rand CS, Powe NR, Wu AW, Wilson MH, Abboud PC, Rubin HR (1999). Why don't physicians follow clinical practice guidelines?. J Am Med Assoc.

[13] Ritter FE, Baxter GD, Churchill EF (2014). Foundations for Designing User-Centered Systems: What System Designers Need to Know About People.

[14] Francke AL, Smit MC, de Veer AJ, Mistiaen P (2008). Factors influencing the implementation of clinical guidelines for health care professionals: a systematic meta-review. BMC Med Inform Decis Mak.

[15] Malterud K, Siersma VD, Guassora AD (2015). Sample size in qualitative interview studies: guided by information power. Qual Health Res.

[16] Morse JM (1995). The significance of saturation. Qual Health Res.

[17] Guest G, Bunce A, Johnson L (2006). How many interviews are enough? An experiment with data saturation and variability. Field Methods.

[18] Veterans Health Administration (2018). VHA Operations Activities That May Constitute Research. Research.va.gov.

[19] Longo L, Kane B (2011). A novel methodology for evaluating user interfaces in health care.

[20] Harrison MB, Légaré F, Graham ID, Fervers B (2010). Adapting clinical practice guidelines to local context and assessing barriers to their use. CMAJ.

[21] List D (2016). The consensus group technique in social research. Field Methods.

[22] Horsky J, Schiff GD, Johnston D, Mercincavage L, Bell D, Middleton B (2012). Interface design principles for usable decision support: a targeted review of best practices for clinical prescribing interventions. J Biomed Inform.

[23] Ivers NM, Sales A, Colquhoun H, Michie S, Foy R, Francis JJ, Grimshaw JM (2014). No more 'business as usual' with audit and feedback interventions: towards an agenda for a reinvigorated intervention. Implement Sci.

[24] Walter Z, Lopez MS (2008). Physician acceptance of information technologies: role of perceived threat to professional autonomy. Decis Support Syst.

[25] Xu Y, Wells PS (2016). Getting (along) with the guidelines: reconciling patient autonomy and quality improvement through shared decision making. Acad Med.

[26] Hess EP, Coylewright M, Frosch DL, Shah ND (2014). Implementation of shared decision making in cardiovascular care: past, present, and future. Circ Cardiovasc Qual Outcomes.

[27] Lewin Group (2014). https://www.healthquality.va.gov/guidelines/CD/lipids/VADoDDyslipidemiaCPG.pdf.

[28] Gagliardi AR, Brouwers MC, Palda VA, Lemieux-Charles L, Grimshaw JM (2011). How can we improve guideline use? A conceptual framework of implementability. Implement Sci.

[29] Damschroder LJ, Robinson CH, Francis J, Bentley DR, Krein SL, Rosland A, Hofer TP, Kerr EA (2014). Effects of performance measure implementation on clinical manager and provider motivation. J Gen Intern Med.

[30] Meyer GS, Nelson EC, Pryor DB, James B, Swensen SJ, Kaplan GS, Weissberg JI, Bisognano M, Yates GR, Hunt GC (2012). More quality measures versus measuring what matters: a call for balance and parsimony. BMJ Qual Saf.

[31] Wright A, Sittig DF, Ash JS, Erickson JL, Hickman TT, Paterno M, Gebhardt E, McMullen C, Tsurikova R, Dixon BE, Fraser G, Simonaitis L, Sonnenberg FA, Middleton B (2015). Lessons learned from implementing service-oriented clinical decision support at four sites: a qualitative study. Int J Med Inform.

[32] Ash JS, Sittig DF, Guappone KP, Dykstra RH, Richardson J, Wright A, Carpenter J, McMullen C, Shapiro M, Bunce A, Middleton B (2012). Recommended practices for computerized clinical decision support and knowledge management in community settings: A qualitative study. BMC Med Infor Decis Mak.

