# Supplementary material for: Designing for Clinical Change: Creating an Intervention to Implement New Statin Guidelines in a Primary Care Clinic
Source: JMIR Hum Factors. 2018 Apr 24;5(2):e19. doi: 10.2196/humanfactors.9030 (PMC5941089; doi:10.2196/humanfactors.9030)
Supplement: Multimedia Appendix 1 [file humanfactors_v5i2e19_app1.pdf]

Multimedia Appendix 1: Single-Page VA/DoD and ACC/AHA Clinical Practice Guidelines Educational Tool.

Concise comparison of VA/DoD and ACC/AHA guidelines for provider education and use.

Risk Category	VA/DoD Clinical recommendation	ACC/AHA guideline
Clinical atherosclerotic cardiovascular disease	If recent, moderate or high-intensity statin.<br>If not recent, moderate- potency statin.	If age ≤75, high-intensity statin. If >75, moderate- potency statin.
Diabetes	Moderate-intensity statin	Moderate-intensity statin
LDL≥190 md/dl	Moderate-intensity statin	Moderate-intensity statin
Risk-based	- ≥12% estimated 10-year risk<br>– Moderate-potency statin<br>- 6-12% estimated 10-year risk<br>– Shared decision-making<br>- <6% estimated 10-year risk<br>– No statin	≥7.5% estimated 10-year ASCVD risk, moderate- or high-potency statin
