# Supplementary material for: Designing for Clinical Change: Creating an Intervention to Implement New Statin Guidelines in a Primary Care Clinic
Source: JMIR Hum Factors. 2018 Apr 24;5(2):e19. doi: 10.2196/humanfactors.9030 (PMC5941089; doi:10.2196/humanfactors.9030)
Supplement: Multimedia Appendix 2 [file humanfactors_v5i2e19_app2.pdf]

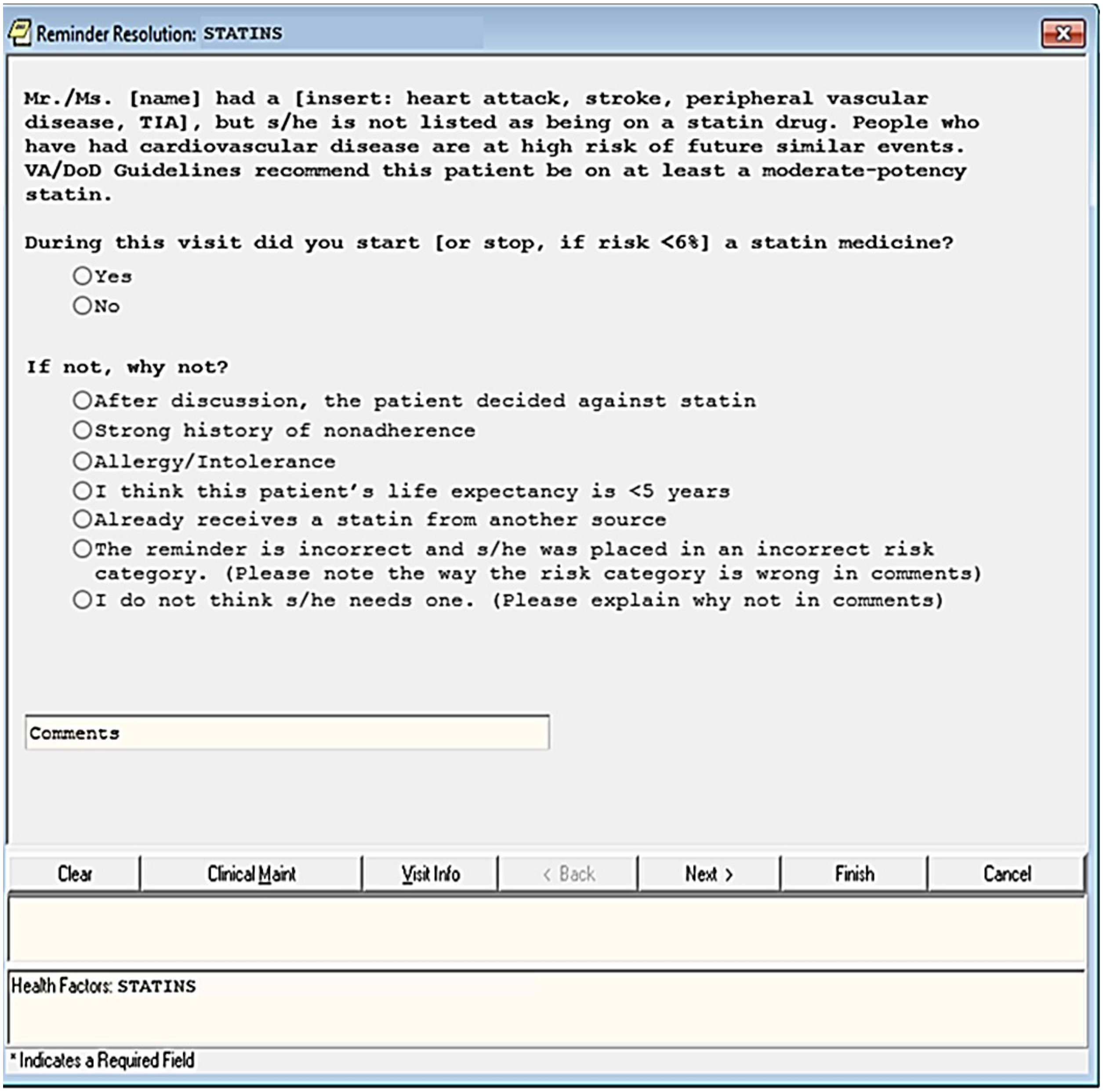

Reminder Resolution: STATINS

Mr./Ms. [name] had a [insert: heart attack, stroke, peripheral vascular disease, TIA], but s/he is not listed as being on a statin drug. People who have had cardiovascular disease are at high risk of future similar events. VA/DoD Guidelines recommend this patient be on at least a moderate-potency statin.

During this visit did you start [or stop, if risk <6%] a statin medicine?

- ○ Yes
- ○ No

If not, why not?

- ○ After discussion, the patient decided against statin
- ○ Strong history of nonadherence
- ○ Allergy/Intolerance
- ○ I think this patient's life expectancy is <5 years
- ○ Already receives a statin from another source
- ○ The reminder is incorrect and s/he was placed in an incorrect risk category. (Please note the way the risk category is wrong in comments)
- ○ I do not think s/he needs one. (Please explain why not in comments)

Comments

Clear | Clinical Maint | Visit Info | < Back | Next > | Finish | Cancel

Health Factors: STATINS

* Indicates a Required Field
