# Supplementary material for: Designing for Clinical Change: Creating an Intervention to Implement New Statin Guidelines in a Primary Care Clinic
Source: JMIR Hum Factors. 2018 Apr 24;5(2):e19. doi: 10.2196/humanfactors.9030 (PMC5941089; doi:10.2196/humanfactors.9030)
Supplement: Multimedia Appendix 3 [file humanfactors_v5i2e19_app3.pdf]

**Risk Category**	**VA/DoD Clinical recommendation**	**HEDIS Performance Measure**	**VA Proposed Performance Measure**
Clinical atherosclerotic cardiovascular disease	Moderate dose statin, consider titrating to high dose as tolerated	Includes	5 points
Diabetes	Moderate dose statin	Includes	3 points
LDL≥190 md/dl	Moderate dose statin	---	1 point
≥ 12% estimated 10-year ASCVD risk	Moderate dose statin	---	1 points
6-12% estimated 10-year ASCVD risk	Shared decision-making	---	Shared-decision-making
<6% estimated 10-year ASCVD risk	No statin	---	1 point if no statin use
