# Supplementary material for: Designing for Clinical Change: Creating an Intervention to Implement New Statin Guidelines in a Primary Care Clinic
Source: JMIR Hum Factors. 2018 Apr 24;5(2):e19. doi: 10.2196/humanfactors.9030 (PMC5941089; doi:10.2196/humanfactors.9030)
Supplement: Multimedia Appendix 4 [file humanfactors_v5i2e19_app4.pdf]

Primary Care Panel: Physician Performance Measurement Report as of <DATE>
<NAME OF PCP>, Medicine Service – General Medicine Section

VA/DOD Clinical Guidelines for Dyslipidemia – Performance Measure Summary

MEASURE	DESCRIPTION	VA GOAL	PATIENTS ELIGIBLE	PATIENTS MEETING	PATIENTS NOT MEETING	% MEETING	PROVIDER PERCENTILE
Statin use: History of ASCVD	Dx: All patients with ASCVD, Age 40-75<br>Goal: Moderate or high dose statin	80%	29	9	20	31%	60
Statin use: Diabetes	Dx: DM, Age 40-75<br>Goal: Moderate dose statin	70%	13	5	8	38%	54
Statin use: LDL ≥190	Dx: LDL ≥190 md/dl, DM, Age 40-75<br>Goal: Moderate dose statin	60%	1	0	1	0%	8
Statin use: High risk	Dx: ≥12% estimated 10-year ASCVD risk, Age 40-75<br>Goal: Moderate dose statin	60%	21	13	8	62%	29
Statin overuse: Low risk	Dx: <4% estimated 10-year ASCVD risk, Age 40-75<br>Goal: No Statin	<10%	11	1	10	9%	83

VA/DOD Clinical Guidelines for Dyslipidemia – Overall Performance Measure Summary

MEASURE	DESCRIPTION	VA GOAL	PATIENTS ELIGIBLE	PATIENTS MEETING	PATIENTS NOT MEETING	% MEETING	PROVIDER PERCENTILE
Overall Statin use	Dx: A) DM (3 points), LDL ≥190 md/dl (1 point), 10-year CVD risk score ≥12 or ASCVD for ages 40-75 (1 point), or all patients with ASCVD (5 points), B) <4% estimated 10-year CVD risk (1 point)<br>Goal: A) Moderate dose statin, B) No statin	>70	1916	707	1209	37%	50
