# Supplementary material for: Designing for Clinical Change: Creating an Intervention to Implement New Statin Guidelines in a Primary Care Clinic
Source: JMIR Hum Factors. 2018 Apr 24;5(2):e19. doi: 10.2196/humanfactors.9030 (PMC5941089; doi:10.2196/humanfactors.9030)
Supplement: Multimedia Appendix 5 [file humanfactors_v5i2e19_app5.pdf]

| Tool | Topic | Suggestion | Impact on adoption |
|---|---|---|---|
| Clinical decision support design | Scenario Language—Add Detail | Include “high/medium/low risk” language in reminder | Implemented |
| | | Use lay language for “elevated ASCVD risk score”— i.e. “elevated risk of heart attack” | Implemented |
| | | Clearly define moderate vs high potency statin | Implemented |
| | | Pre-populate risk score automatically within reminder | Future consideration |
| | | Add links to guidelines and background information | Future consideration |
| | | Add specific risk percentage in reminder rather than “high/medium/low” language | Not used |
| | Reminder Algorithm | Disable reminder for patients receiving palliative care | Implemented |
| | | Only alert when appropriate (disable for patients with complicated clinical situations) | Future consideration |
| | | Add additional pop-up reminder for comments | Not used |
| | Answer Choices | Add “receiving statin from other source” option | Implemented |
| | | Add “did not have time to address at this visit” option | Future consideration |
| | | Remove “hx of nonadherence” option | Not used |
| | Helpful Additions | “Order now” option | Future consideration |
| | | Direct link to risk calculator | Future consideration |
| | | Add link to algorithm | Future consideration |
| Audit and feedback design | Goals/Targets | Clarify provider comparison group (local vs. VA) | Future consideration |
| | | Remove provider percentile altogether because it creates undue angst | Not used |
| | | Standardize goal percentages across categories | Not used |
| | A&F Algorithm | Provide credit for shared decision making | Future consideration |
| | | Devise mechanism/algorithm that accounts for complicated patients in performance measure and subsequently in the audit and feedback report | Future consideration |
| | Actionable Feedback | Organize patient “fallout” by risk category | Implemented |
| | | Include specific and actionable performance improvement suggestions | Future consideration |
| | Simplify Language | Replace men/women with M/F | Implemented |
| General suggestions | Educational opportunities | Journal club/ educational session requested when new guidelines emerge | Future consideration |
